# Gender Differences in a Mouse Model of Hepatocellular Carcinoma Revealed Using Multi-Modal Imaging

**DOI:** 10.3390/cancers15153787

**Published:** 2023-07-26

**Authors:** Brian J. Engel, Vincenzo Paolillo, Md. Nasir Uddin, Kristyn A. Gonzales, Kathryn M. McGinnis, Margie N. Sutton, Madhavi Patnana, Brian J. Grindel, Gregory J. Gores, David Piwnica-Worms, Laura Beretta, Federica Pisaneschi, Seth T. Gammon, Steven W. Millward

**Affiliations:** 1 Department of Cancer Systems Imaging, UT MD Anderson Cancer Center, Houston, TX 77030, USA; 2 Cyclotron Radiochemistry Facility, UT MD Anderson Cancer Center, Houston, TX 77030, USA; 3 Department of Molecular and Cellular Oncology, UT MD Anderson Cancer Center, Houston, TX 77030, USA; 4 Department of Abdominal Imaging, UT MD Anderson Cancer Center, Houston, TX 77030, USA; 5 College of Medicine, The Mayo Clinic, Rochester, MN 55905, USA; 6 Center for Translational Cancer Research, The Brown Foundation Institute of Molecular Medicine for the Prevention of Human Diseases (IMM) at the University of Texas Health Science Center at Houston, Houston, TX 77030, USA

**Keywords:** NALFD, PET, NASH, HCC, apoptosis, inflammation, MRS

## Abstract

**Simple Summary:**

The worldwide prevalence of liver cancer, an extremely lethal disease, continues to increase. Although there are many risk factors associated with liver cancer including a fatty liver, metabolic syndrome (diabetes), and lifestyle choices (poor diet and alcohol abuse), it is still challenging to predict which patients are most likely to develop the disease and identify the most effective interventions. In this study, we carried out a non-invasive, multi-modal study of liver cancer in a mouse model to identify imaging characteristics (imaging biomarkers) that could be used to predict the development of malignant disease. We found that multiple clinical imaging techniques could predict liver cancer development in male mice, while only a handful of imaging techniques had any predictive power in female mice. The results of this study provide evidence that imaging biomarkers can predict liver cancer in mouse models and that mouse gender can significantly influence liver tumor development.

**Abstract:**

The worldwide incidence of hepatocellular carcinoma (HCC) continues to rise, in part due to poor diet, limited exercise, and alcohol abuse. Numerous studies have suggested that the loss or mutation of PTEN plays a critical role in HCC tumorigenesis through the activation of the PI3K/Akt signaling axis. The homozygous knockout of PTEN in the livers of mice results in the accumulation of fat (steatosis), inflammation, fibrosis, and eventually progression to HCC. This phenotype bears a striking similarity to non-alcoholic steatohepatitis (NASH) which is thought to occupy an intermediate stage between non-alcoholic fatty liver disease (NAFLD), fibrosis, and HCC. The molecular and physiological phenotypes that manifest during the transition to HCC suggest that molecular imaging could provide a non-invasive screening platform to identify the hallmarks of HCC initiation prior to the presentation of clinical disease. We have carried out longitudinal imaging studies on the liver-specific PTEN knockout mouse model using CT, MRI, and multi-tracer PET to interrogate liver size, steatosis, inflammation, and apoptosis. In male PTEN knockout mice, significant steatosis was observed as early as 3 months using both magnetic resonance spectroscopy (MRS) and computed tomography (CT). Enhanced uptake of the apoptosis tracer ^18^F-TBD was also observed in the livers of male PTEN homozygous knockout mice between 3 and 4 months of age relative to heterozygous knockout controls. Liver uptake of the inflammation tracer [^18^F]4FN remained relatively low and constant over 7 months in male PTEN homozygous knockout mice, suggesting the suppression of high-energy ROS/RNS with PTEN deletion relative to heterozygous males where the [^18^F]4FN liver uptake was elevated at early and late time points. All male PTEN homozygous mice developed HCC lesions by month 10. In contrast to the male cohort, only 20% (2 out of 10) of female PTEN homozygous knockout mice developed HCC lesions by month 10. Steatosis was significantly less pronounced in the female PTEN homozygous knockout mice relative to males and could not accurately predict the eventual occurrence of HCC. As with the males, the [^18^F]4FN uptake in female PTEN homozygous knockout mice was low and constant throughout the time course. The liver uptake of ^18^F-TBD at 3 and 4.5 months was higher in the two female PTEN knockout mice that would eventually develop HCC and was the most predictive imaging biomarker for HCC in the female cohort. These studies demonstrate the diagnostic and prognostic role of multi-modal imaging in HCC mouse models and provide compelling evidence that disease progression in the PTEN knockout model is highly dependent on gender.

## 1. Introduction

The incidence of liver cancer is rising throughout the world and is the fourth most common cause of cancer mortality behind lung, colorectal, and stomach cancer [1]. Hepatocellular carcinoma (HCC) comprises 75–85% of all liver cancers, with intrahepatic cholangiocarcinoma and other rare types comprising the remainder of cases [1]. Although the etiology of HCC is complex, the fraction of HCC cases resulting from metabolic syndrome, cirrhosis from alcohol abuse, chronic hepatitis B or C infections, non-alcoholic steatohepatitis (NASH), or non-alcoholic fatty liver disease (NAFLD) is rapidly increasing [2,3]. HCC is associated with poor prognosis, with a 41–75% 5-year survival rate following primary tumor resection in non-cirrhotic patients and a 30–50% 5-year survival rate in cirrhotic patients [4]. HCC disproportionately affects males with a male:female ratio of 2–3 for both incidence and mortality rates [5]. While progress has been made in the development of biomarkers to predict prognosis and response to therapy [6,7], selective and sensitive biomarkers for population screening remain elusive [8]. Although HCC is an aggressive and lethal disease, it is generally thought to pass through a series of one or more non-malignant liver pathologies, including NAFLD, non-alcoholic steatohepatitis (NASH), fibrosis, and cirrhosis [9]. This multi-year continuum represents an opportunity to identify increased HCC risk early and to intervene before liver damage becomes irreversible and HCC becomes inevitable. 

The loss of the phosphatase and tensin homolog deleted on the chromosome 10 (PTEN) tumor suppressor has been shown to play a key role in the development of HCC. The loss of heterozygosity (LOH) at the PTEN locus is observed in approximately 30% of HCC tumors [10], and PTEN expression is reduced in approximately 40% of HCC tumors [11,12]. PTEN catalyzes the dephosphorylation of phosphatidylinositol-3,4,5-trisphosphate (PIP_3_) to phosphatidylinositol-4,5-bisphosphate (PIP_2_), which prevents membrane localization and the activation of Akt (PKB) kinases [13]. The dysregulation of Akt signaling results in excessive pro-growth, pro-inflammatory, and anti-apoptotic signaling [14]. The nuclear translocation of PTEN results in a cell cycle arrest, activation of DNA repair proteins, and pro-apoptotic signaling—effects that are attenuated when PTEN activity is reduced or eliminated [15]. 

Selective PTEN deletion has been shown to induce tumor formation in murine models of cancer [16,17,18]. Selective homozygous deletion of the PTEN gene in the liver of mice results in dramatically increased liver steatosis (increased liver fat), initiation of fibrosis, inflammation, and eventual progression to HCC at 10 months of age [19]. This model also recapitulates some of the characteristics of NASH, including steatosis and inflammation, and therefore represents a tool for developing interventions to inhibit progression to cirrhosis and fibrosis. As is the case of HCC in the human population, the PTEN deletion model reveals a significant gender imbalance—HCC development is nearly 100% penetrant in male mice and <50% penetrant in female mice [20]. Previous work on the liver-specific PTEN deletion model has focused on the HCC endpoint, but very few data exist to define the molecular and physiological milestones that manifest prior to the emergence of malignant disease. The role of gender in this model is also unclear, although recent studies have shown that androgen receptor phosphorylation and nuclear translocation driven by excessive Akt signaling in hepatocytes profoundly influence HCC progression [21]. 

To gain mechanistic insight, we undertook a longitudinal characterization of liver steatosis, volume, apoptosis, and inflammation of the liver-specific PTEN deletion mouse model of HCC between 3 and 10 months of age. A multi-modal, non-invasive imaging strategy was chosen for analysis, thereby allowing each animal to serve as its own comparator control over time. Computed tomography (CT) and magnetic resonance spectroscopy (MRS) were used to assess steatosis. MRI and positron emission tomography (PET) were used to measure changes in liver volume. The PET radiotracers ^18^F-TBD [22] and [^18^F]4FN [23] were used to measure the extent of apoptosis and high-energy reactive oxygen species/reactive nitrogen species (ROS/RNS), respectively. These longitudinal studies revealed significant differences in the onset and trajectory of steatosis and hepatomegaly between male and female PTEN deletion mice. Elevated liver uptake of ^18^F-TBD was observed in PTEN homozygous knockout male mice at early time points and was found to be modestly predictive for HCC in the female cohort. In contrast, homozygous PTEN deletion was associated with suppressed high-energy ROS/RNS in both male and female mice. These studies provide quantitative imaging data to substantiate the role of gender in the PTEN-driven HCC mouse models and suggest new strategies for the prediction of HCC and therapeutic intervention.

## 2. Materials and Methods

### 2.1. Radiochemistry of ^18^F-TBD

Preparation of the stripping resin: A glass column (Bio-Rad Econo-Column 1.5 × 5 cm) was filled with approximately 500 mg of TG-AcN_3_ resin synthesized in-house following a procedure reported previously [24]. The resin was washed with 60% (*v*/*v*) ethanol in water and equilibrated for 1 h with a solution of stripping buffer. Stripping buffer was prepared by mixing 620 mg of CuSO_4_ pentahydrate, 4.4 g of L-ascorbic acid, 200 mL of deionized water, 10 mL of glacial acetic acid, 300 mL of 200 proof, and anhydrous ethanol (pre-treated for 5 min with 150 mg of p-toluensulfonyl hydrazine resin). The solution was stirred for 5 min and then filtered under a vacuum. Fresh stripping resin was prepared prior to each radiosynthesis.

Radiosynthesis of ^18^F-TBD: Radiosynthesis of ^18^F-TBD was performed using a TRACERlab FN-FX automatic module (General Electric Healthcare, Münster, Germany). [^18^F]Fluoride was obtained as an aqueous solution from the MD Anderson Cyclotron Radiochemistry Facility (CRF). [^18^F]Fluoride was adsorbed on an ion exchange cartridge (pre-conditioned Sep-PAK^®^ Light QMA Cartridge, ABX GmbH, Radeberg, Germany). [^18^F]Fluoride was flushed into Reactor 1 with a potassium carbonate and Kryptofix 2.2.2. water/CH_3_CN solution (700 μL; 52.8 mg of K_2_CO_3_, 240.1 mg of K_222_, 4 mL of water, 16 mL of CH_3_CN) contained in Vial 1. The solution was dried under a vacuum and nitrogen flow at 80 °C for 2 min. Reactor 1 was cooled to 60 °C, and dry acetonitrile (500 μL) was added from Vial 2. The mixture was azeotropically dried at 120 °C for 2 min. The tosyl fluoroethyl azide precursor (10 μL) in dry acetonitrile (500 µL) was added into Reactor 1 through Vial 3. The mixture was stirred at 90 °C for 15 min. Reactor 1 was cooled, and [^18^F]Fluoroethylazide was then distilled for 5 min at 60 °C into a vial containing a “click” mixture of CuSO_4_ pentahydrate (50 μL; 35 mg/mL in water), sodium ascorbate (50 μL; 174 mg/mL in PBS), TBTA (13 μL; 100 mg/mL in DMF), TBD-alkyne precursor (1.7 μmol in 87 μL of DMF), and piperidine (20 μL; 20% (*v*/*v*)). The “click” mixture was previously prepared by mixing CuSO_4_ pentahydrate and sodium ascorbate until the color of the solution turned milky white, then adding the other reagents in turn.

The click reaction was performed for 10 min at room temperature, then quenched with the stripping buffer (1 mL) delivered from Vial 7. The mixture was transferred into the stripping column and eluted with the stripping buffer at 0.2 mL/min. Radioactivity came off the resin between 8 and 25 min and was collected in the quenching vial containing 0.17% (*v*/*v*) of phosphoric acid (7.5 mL). The solution was then passed onto a Sep-Pack C18 light cartridge, pre-conditioned with ethanol (3 mL) and water (6 mL), and the radioactivity was eluted off the cartridge with a 1:1 mixture of ethanol and PBS, then diluted with PBS to reach a final ethanol concentration of 10%. Dilution in PBS caused precipitation of a white solid—likely TBTA—that was filtered off a Millex GV 0.22 μm sterile filter.

Activity was determined with a dose calibrator (Capintec, Mirion Technology, Florham Park, NJ, USA), and a sample was taken for quality control (QC). QC was performed with analytical radio-HPLC (Agilent 1260 infinity II, Santa Clara, CA, USA) using a C18 column (Phenomenex Inc., Gemini 5u, 150 × 4.6 mm, Torrance, CA, USA, 1 mL/min flow rate) and a water (A)/acetonitrile 0.1% TFA(B) gradient (B: 5% for 1 min, 5–60% for 15 min, 60–95% for 1 min, and 95% for 2 min). The identity of ^18^F-TBD was confirmed by co-elution with ^19^F-TBD synthesized in-house. ^18^F-TBD was obtained in 7.2 ± 3.4% yield (non-decay corrected) and 99% radiochemical purity (N = 16) (Figure S1C).

Radiochemistry of [^18^F]4FN: [^18^F]4FN was synthesized following the procedure reported previously [23].

### 2.2. Dynamic PET Imaging with ^18^F-TBD

Mice were lightly anesthetized and placed in the pre-clinical imaging system. A 30 min list mode acquisition (15 cm axial FOV) was started, and ^18^F-TBD was injected just after the acquisition started. A single-station CT was automatically acquired after the 30 min acquisition. Daily PET QC was acquired with a Ge-68 flood phantom and at least an annual quantitative calibration. PET dynamic data were reconstructed with MLEM-3D, 12 iterations, using decay random and scatter correction. Volumes of interest for the liver were guided using CT and defined with iso-contour analysis of the peak liver uptake of ^18^F-TBD (PMOD). A 3 mm VOI centered on the heart (CT-guided) was used to measure the heart at each time point. Liver-to-heart ratios (as representative of blood pool counts) were calculated. Unlike previous reports, visual and quantitative contrast were discernable in the liver/heart ratio obviating more complex kinetic modeling [22]. 

### 2.3. PET Imaging with [^18^F]4FN and [^18^F]FDG

Mice were injected with a target of 100 μCi (3.7 MBq) per mouse with either of the tracers, and data were corrected on a per-mouse basis for the actual injected dose. Mice were imaged with a 10 min PET with a 15 cm axial field-of-view (FOV) acquired at 1 h post-injection for [^18^F]4FN or 40 min post-injection for [^18^F]Fluorodeoxyglucose (FDG). After each PET scan, a liver-centered CT, with 7 cm axials, was automatically acquired. PET data were reconstructed with MLEM-3D using 12 iterations, and CT data were reconstructed with FBP. Daily PET QC was acquired with a flood phantom and at least an annual quantitative calibration. For [^18^F]FDG PET scans, mice were diet-restricted with ad libitum water at least 3 h prior to data acquisition, with the cage kept on a warming pad. Post-scan food was returned to the cage. For [^18^F]4FN acquisitions, no dietary changes were required.

Representative 3 cm radius regions of interest (ROIs) were drawn within the liver guided using CT for both the liver and heart as a representative of the blood pool after confirming generally homogeneous uptake within the liver (true for all livers at all time points in the PTEN model). 

### 2.4. Quantitation of Liver Fat and Liver Volume Using MR

For all MR imaging liver fat experiments, animals were anesthetized with 2% isoflurane. Imaging was conducted in a 4.7 T small animal MR scanner (Biospec, Bruker Biospin MRI, Inc., Billerica, MA, USA) using transmit/receive volume coils with 35 mm inner diameter. The following parameters were used for all sequences: a field-of-view (FOV) of 4 × 3 cm^2^, a matrix of 256 × 192, a spatial resolution of 156 μm, and a T2-weighted axial and coronal rapid acquisitions with relaxation enhancement (RARE) acquisition (echo time, 57 ms; repetition time, 4000 ms; slice thickness, 0.75 mm; slice gap, 0.25 mm). Four voxels of 0.292 mm × 0.677 mm × 0.677 mm were placed in the upper left, upper right, lower left, and lower right of the acquired T2-weighted scans in the liver to calculate the average of the fat percentages in mice (PRESS scan; echo time, 14.9 ms; repetition time, 10,000 ms). Analysis was performed using ImageJ ver 1.54 software.

### 2.5. Measurement of Liver Density Using CT

A representative 3 mm radius ROI was placed in the liver using PMOD (PMOD Technologies, Fällanden, Switzerland), and the average density (HU) was obtained for each mouse at each time point. These data were acquired as part of the [^18^F]4FN PET/CT imaging experiments. 

### 2.6. PTEN Deletion Mouse Model

All animal procedures were carried out in accordance with the policies and regulations of the Institutional Animal Care and Use Committee (IACUC) at the University of Texas MD Anderson Cancer Center. Experimental mice were *Pten*^loxP/loxP^; Albumin-Cre^+^ that were obtained through the crossing of *Pten*^loxP/loxP^; Albumin-Cre^+^ male mice and *Pten*^loxP/+^; Albumin-Cre^+^ female mice (C57BL/6 background). This resulted in selective homozygous or heterozygous deletion of a significant portion of the *Pten* gene in hepatocytes [19]. Mice were purchased from Jackson Labs and given normal chow throughout the course of the longitudinal study. Pathology and immunohistochemistry were carried out with HistoWiz (Brooklyn, NY, USA). In the longitudinal study [^18^F-TBD (PET), [^18^F]4FN (PET), %Liver Fat (MRS), Liver Density (CT), and liver volume (PET)], five PTEN homozygous KO male mice, five PTEN heterozygous KO male mice, and ten PTEN homozygous KO female mice were used. One PTEN homozygous KO female died between 8 and 10 months (unrelated to imaging) and therefore was not considered in the 10-month data set. In the [^18^F]FDG PET and ^18^F-TBD PET validation imaging experiments, a separate cohort of five PTEN heterozygous KO males and six PTEN homozygous KO males were imaged at 3 and 4.5 months of age.

## 3. Results

### 3.1. Liver PTEN Model—Males

Transgenic mice where the PTEN gene was selectively knocked out in the liver were bred as described previously [19]. For male mice, two cohorts were examined in this study. In the first male cohort, both copies of the PTEN gene were deleted (PTEN^−/−^), while in the second male cohort, only one copy of the gene was deleted (PTEN^+/−^). The PTEN^−/−^ group was predicted to progress through a NASH-like phenotype to HCC, while the PTEN^+/−^ group was predicted to retain a normal liver phenotype throughout the time course. Beginning at 3 months of age, both male cohorts were imaged using PET/CT (^18^F-TBD and [^18^F]4FN) as well as MRI/MRS (% liver fat with MRS, liver volume with MRI). Mice were re-imaged at 4.5, 6, 8, and 10 months. Following the sacrifice at the 10-month time point, all PTEN^−/−^ males showed the expected HCC phenotype as defined by a total tumor volume greater than 7.5 mm^3^. As expected, none of the PTEN^+/−^ males showed HCC tumors at 10 months. 

**Figure 1:**
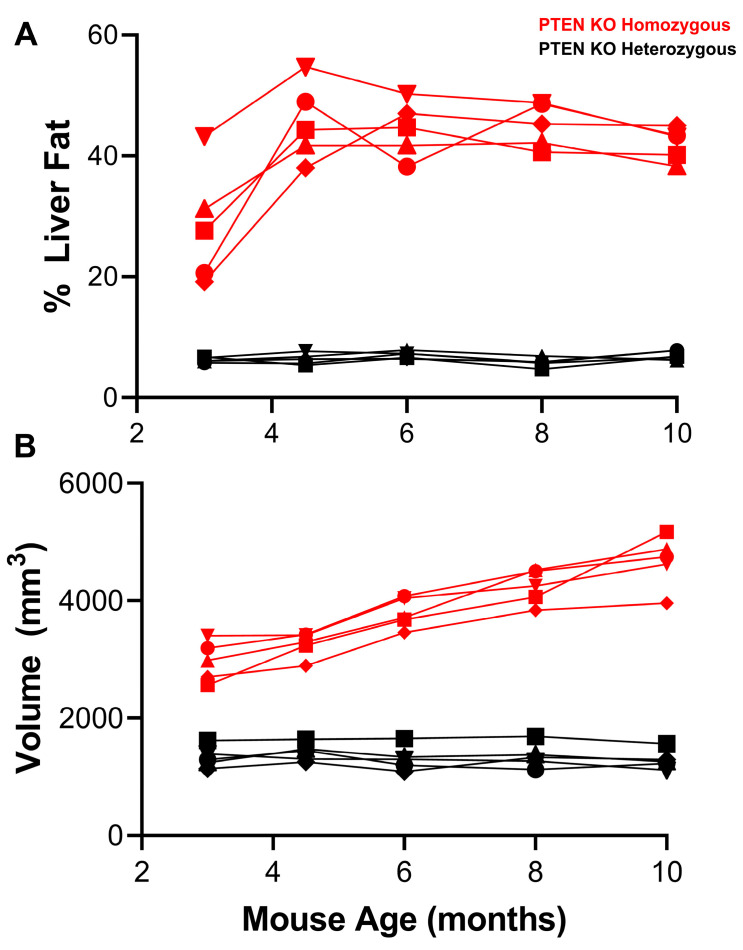
Steatosis and liver volume in male mice. (**A**) % liver fat was measured with MRS and (**B**) liver volume (mm^3^) was measured with MRI in the PTEN KO homozygous group (red) and PTEN KO heterozygous group (black) from 3 months to 10 months.

The extent of liver steatosis was assessed by measuring the % liver fat using MRS [25]. As seen in Figure 1A, the % liver fat remained effectively unchanged in the PTEN^+/−^ group throughout the time course of the study (~4–8%). In contrast, the % liver fat in the PTEN^−/−^ group was significantly higher than the control group as early as 3 months of age and plateaued at approximately 40% liver fat by 4.5 months. The measurement of liver density using CT revealed a similar pattern with PTEN^−/−^ male mice showing significantly lower liver densities relative to PTEN^+/−^ male mice at the earliest time point (Figure S2A). Subsequent histopathological analysis of an independent cohort of PTEN homozygous KO and heterozygous KO male mice (4–4.5 months) confirmed highly steatotic livers in the PTEN^−/−^ group relative to the control group (Figure S3).

Liver volumes in the male PTEN^+/−^ group measured with MRI [26] remained very constant over the time course (approximately 1500 mm^3^) (Figure 1B). In contrast, liver volume increased in a linear fashion in the PTEN^−/−^ group from approximately 3000 mm^3^ at 3 months to >4000 mm^3^ at 10 months. The measurement of liver volume with PET (using ^18^F-TBD) showed a similar trend in both cohorts albeit with significantly lower precision (Figure S2B). Increased liver size (hypermegaly) and increased steatosis are both hallmarks of the PTEN model [19] and were readily apparent in this study.

**Figure 2:**
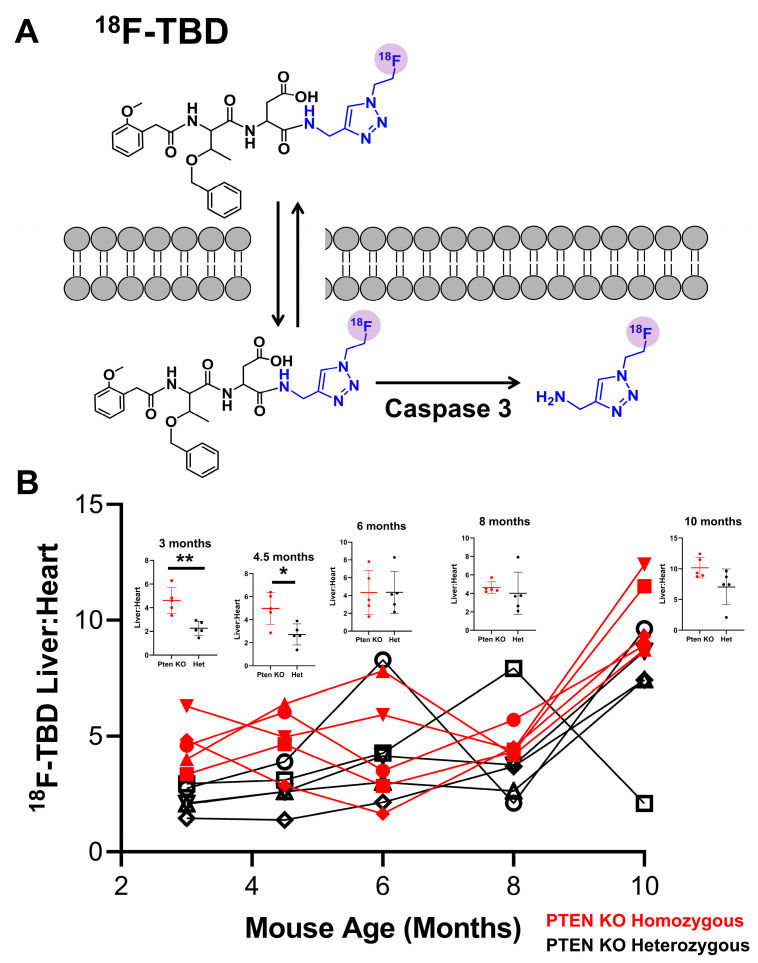
Liver uptake of the apoptosis tracer ^18^F-TBD in male mice. (**A**) In apoptotic cells, ^18^F-TBD is cleaved in the presence of caspase 3, resulting in the formation of a hydrophilic fragment bearing the F18 radiolabel. (**B**) Liver:heart ratio of ^18^F-TBD activity at 30 min post-injection in PTEN KO homozygous and PTEN KO heterozygous male mice (* *p* ≤ 0.05, ** *p* ≤ 0.01, Student’s *t*-test).

We next sought to determine the extent of apoptosis and immune infiltration resulting from homozygous PTEN deletion in the liver. To interrogate apoptosis, we employed the PET radiotracer ^18^F-TBD. This probe was selectively cleaved with caspase 3 and was previously shown to result in the accumulation of activity in response to hepatocellular apoptosis [22] (Figure 2A). The original synthesis of ^18^F-TBD described by Engel and coworkers [22] was implemented to achieve a fully automated protocol. In the previous method, [^18^F]fluoroethylazide was conjugated to the alkyne precursor through the copper-catalyzed azide–alkyne cycloaddition. The resulting ^18^F-TBD tracer was purified by scavenging the alkyne precursor on a TentaGel-based azide resin [24]. Although this resin was integrated into the TRACERlab fluid path, the introduction of the cartridge and elution of the product from this resin were performed semi-manually. Since a study like the one reported herein needed higher doses of ^18^F-TBD for multiple animals’ PET imaging sessions, we elected to incorporate the azide resin as a fully automated component in the radiosynthetic workflow. A TRACERlab FN-FX automatic module was modified to accommodate the column containing the resin that replaced the standard HPLC column (Figure S1A,B). The injector was bypassed in the final TRACERlab design.

Both cohorts of male mice were injected with approximately 100 µCi of ^18^F-TBD (i.v.) and imaged with a dynamic PET/CT for 30 min. Although time–activity data were collected for multiple physiological compartments during these studies, a relatively simple liver:heart ratio obtained at 30 min post-injection was sufficient to discriminate between the PTEN KO homozygous and heterozygous groups (Figure 2B). Indeed, the liver:heart of ^18^F-TBD uptake was approximately two-fold higher in the PTEN^−/−^ male group relative to the PTEN^+/−^ male group at both 3 and 4.5 months of age. The differences in the liver uptake of ^18^F-TBD between the groups all but disappeared between 4.5 months and 8 months of age. At the terminal 10-month time point, we again observed higher liver uptake of ^18^F-TBD in the PTEN^−/−^ group, although this failed to reach the level of statistical significance. The histopathological analysis of an independent cohort of PTEN homozygous KO and heterozygous KO male mice (4–4.5 months) revealed no difference in liver apoptosis with cleaved caspase 3 or TUNEL staining, suggesting that increased ^18^F-TBD liver uptake was not the result of increased apoptosis (Figures S4 and S5). 

**Figure 3:**
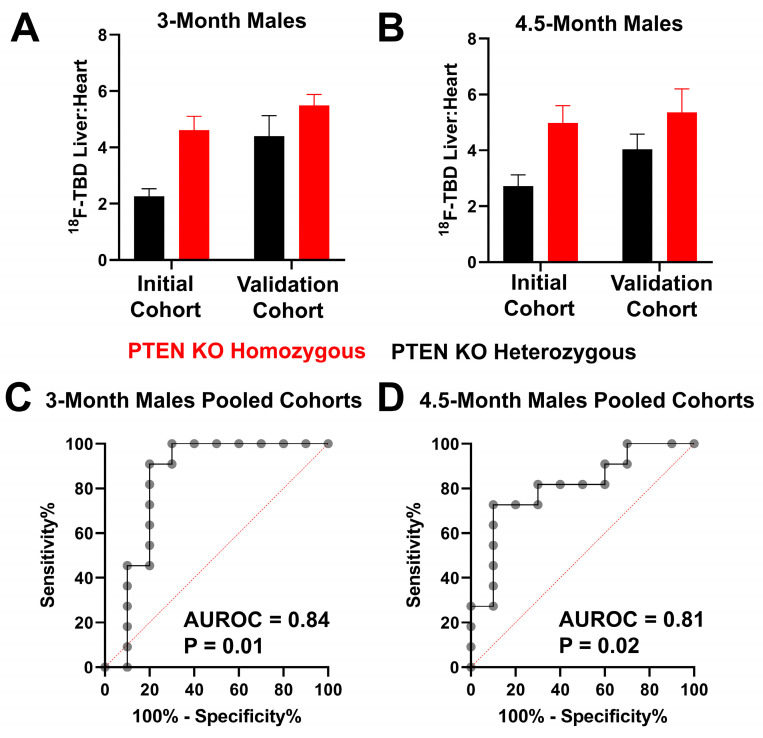
Validation of enhanced ^18^F-TBD liver uptake in male PTEN KO homozygous mice. ^18^F-TBD liver:heart at (**A**) 3 and (**B**) 4.5 months in PTEN KO homozygous and heterozygous male mice in the initial study (initial cohort) and validation study (validation cohort). Receiver–operator analysis of (**C**) pooled 3-month data and (**D**) pooled 4.5-month data.

Although the mechanism of enhanced ^18^F-TBD liver uptake in the PTEN^−/−^ male cohort remains unclear, the longitudinal study suggested that it may provide a useful molecular imaging biomarker to predict HCC months before the onset of the disease. To verify this observation, we carried out a validation study with five male PTEN^−/−^ and five male PTEN^+/−^ mice at 3 and 4.5 months of age. These experiments were carried out more than one year after the initial longitudinal study at these timepoints. As seen in Figure 3A,B, PTEN^−/−^ male mice in the validation cohort once again showed enhanced ^18^F-TBD liver:heart relative to PTEN^+/−^ mice at 3 and 4.5 months of age. The integration of the two datasets followed by a receiver–operator analysis reveals significant predictive power at both 3 (AUROC = 0.84) and 4.5 months (AUROC = 0.81) (Figure 3C,D). 

**Figure 4:**
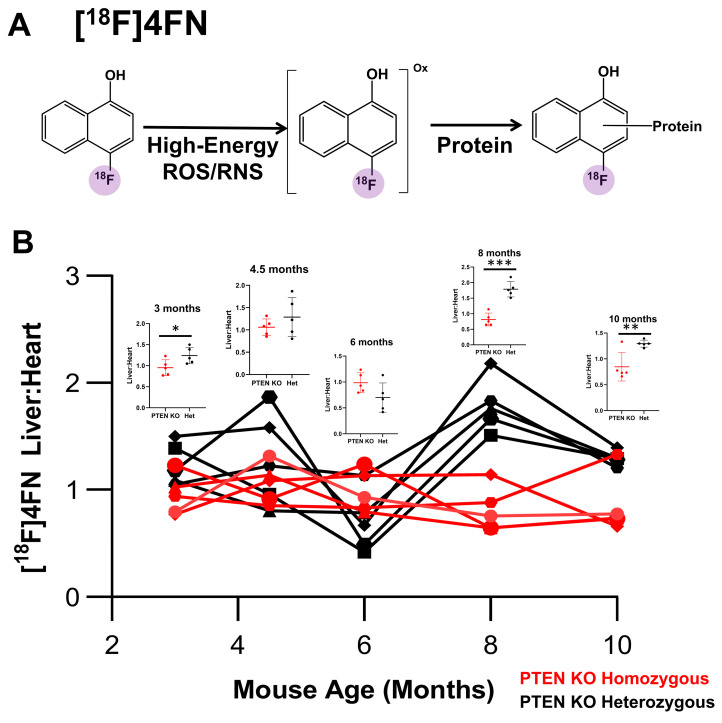
Liver uptake of [^18^F]4FN in male mice. (**A**) Oxidation of [^18^F]4FN with high-energy ROS/RNS at the site of immune infiltration and inflammation results in a reactive intermediate, which is believed to covalently react with endogenous radical acceptors such as proteins, resulting in trapping and accumulation of activity. (**B**) Liver:heart ratio of [^18^F]4FN activity at 60 min post-injection in PTEN KO homozygous (red) and PTEN KO heterozygous (black) male mice. (* *p* ≤ 0.05, ** *p* ≤ 0.01, *** *p* ≤ 0.001, Student’s *t*-test).

Increased inflammation and immune infiltration in the liver are associated with the initiation and development of HCC [27]. As such, we sought to use [^18^F]4FN to evaluate the presence of high-energy ROS/RNS in the livers of PTEN^−/−^ and heterozygous male mice (Figure 4A). Both groups of male mice were injected with approximately 100 µCi of [^18^F]4FN and imaged using PET/CT after 1 h (Figure 4B). As with ^18^F-TBD, the ratio of uptake between the liver and heart at 30 min post-injection provided a useful, normalized measurement of tracer uptake. Strikingly, [^18^F]4FN liver uptake in the male PTEN^−/−^ group was remarkably low and constant throughout the time course of the study. In contrast, the [^18^F]4FN liver uptake in the male PTEN^+/−^ group varied significantly with statistically significant increases relative to the PTEN^−/−^ groups seen at 3 months and late in the time course (months 8–10). The histopathological analysis of an independent cohort of PTEN homozygous KO and heterozygous KO male mice (4–4.5 months) indicated low myeloperoxidase protein content and macrophage/dendritic cell infiltration in both cohorts (Figures S6 and S7). Taken together, these data suggested that PTEN deletion may result in the suppression of immune infiltration and high-energy ROS/RNS activity in the liver.

**Figure 5:**
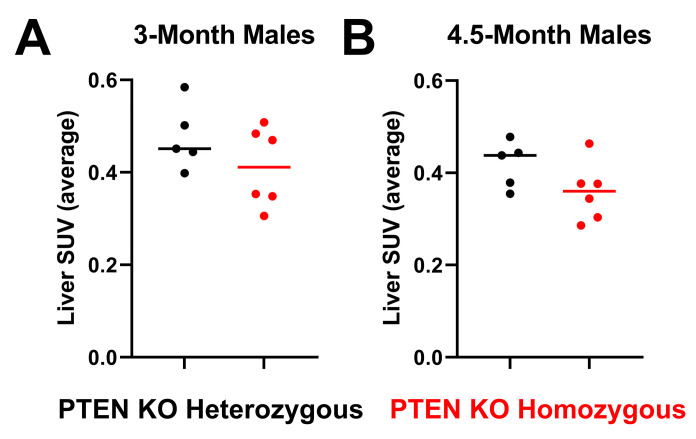
Liver uptake of [^18^F]-FDG in male mice. [18F]-FDG liver uptake (SUVaverage) in PTEN KO homozygous and heterozygous male mice at (**A**) 3 months and (**B**) 4.5 months of age.

Given the power of most imaging biomarkers in this study to discriminate between PTEN^−/−^ and PTEN^+/−^ male mice, it was of interest to determine if [^18^F]FDG, the most commonly used PET radiotracer, showed similar predictive power in this model. As such, we carried out standard [^18^F]FDG PET/CT imaging experiments (static imaging, 1 h post-injection) on male mice at 3 and 4.5 months of age (Figure 5). [^18^F]FDG liver uptake as measured using SUV_AVG_ was low in both groups, and there was no statistically significant difference in the liver uptake between male PTEN^−/−^ and male PTEN^+/−^ mice. As expected, the receiver–operator analysis revealed no significant discriminatory power of [^18^F]FDG at these time points.

### 3.2. Liver PTEN Model—Female Cohort

The homozygous deletion of PTEN in male mice results in complete phenotypic conversion to HCC in 10 months. In contrast, homozygous deletion of PTEN in the livers of female mice is characterized by incomplete penetrance, with some reports indicating that the complete PTEN deletion results in HCC in only 50% of the female population [20]. We sought to (1) monitor the development of HCC in PTEN^−/−^ female mice using the imaging modalities described above and (2) assess their ability to predict which individuals in a PTEN KO homozygous female population will eventually progress to HCC. In this study, ten female PTEN^−/−^ mice were monitored over the course of 7 months. Only two of these mice developed HCC as defined by a total tumor burden in the liver of ≥7.5 mm^3^. These mice are indicated in red in the subsequent figures, although their HCC status was only determined at the terminal 10-month time point.

**Figure 6:**
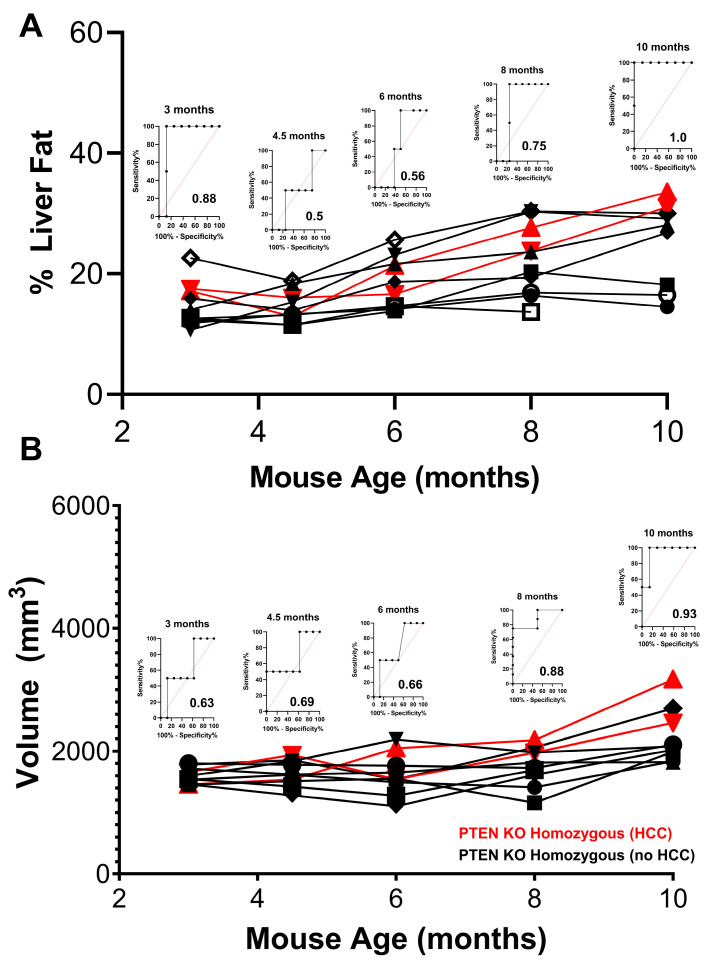
Image-based measurement of steatosis and liver volume in PTEN KO homozygous female mice. (**A**) % liver fat in female PTEN KO homozygous mice from 3–10 months of age (red = HCC progressor, black = HCC non-progressor). (**B**) Liver volume in female PTEN KO homozygous mice from 3–10 months of age (red = HCC progressor, black = HCC non-progressor). ROC analyses at each time point are inset.

The changes in liver size and steatosis in a group of female PTEN^−/−^ mice were monitored using MRI and MRS as described above. As seen in Figure 6A, the mean % liver fat at the initial 3-month time point was approximately 20%, which was lower than the PTEN^−/−^ male group at the same time point (~30%) but higher than the PTEN^+/−^ male group at 3 months (~5%). In contrast to the male PTEN^−/−^ cohort, the increase in median liver fat over the course of the study in the female group was significantly slower, reaching only ~20% after 7 months. This imaging biomarker showed a significant predictive power for eventual HCC development at 3 months (Area Under the Receiver Operator Curve, AUROC = 0.88) but was relatively non-predictive throughout the rest of the study until 10 months. At no point in the study was there a dramatic difference in liver fat between HCC progressors and non-progressors. Conversely, this difference was significant in the male cohort as early as 3 months and became more pronounced as the mice grew older. CT imaging corroborated the MRS-based analysis of liver fat and showed normal liver densities at 3 months (median of 8 HU), which decreased modestly throughout the study (Figure S8A).

The liver size in the PTEN^−/−^ female cohort showed a similar pattern. The liver size for all female PTEN^−/−^ mice was normal at 3 months (1500–2000 mm^3^) and remained effectively constant from months 3–8 (Figure 6B). A slight increase in median liver size was observed in month 10, which was significantly (although not perfectly) predictive of HCC progression. The liver size was almost completely unpredictive for HCC progression from 3–6 months. The measurement of liver size with PET corroborated these observations, although the two HCC-progressing female mice showed large increases in liver volume between 6–10 months of age (Figure S8B). In summary, the changes in the liver size of female PTEN^−/−^ mice over time were quite modest and more akin to the PTEN^+/−^ males rather than the PTEN^−/−^ males.

**Figure 7:**
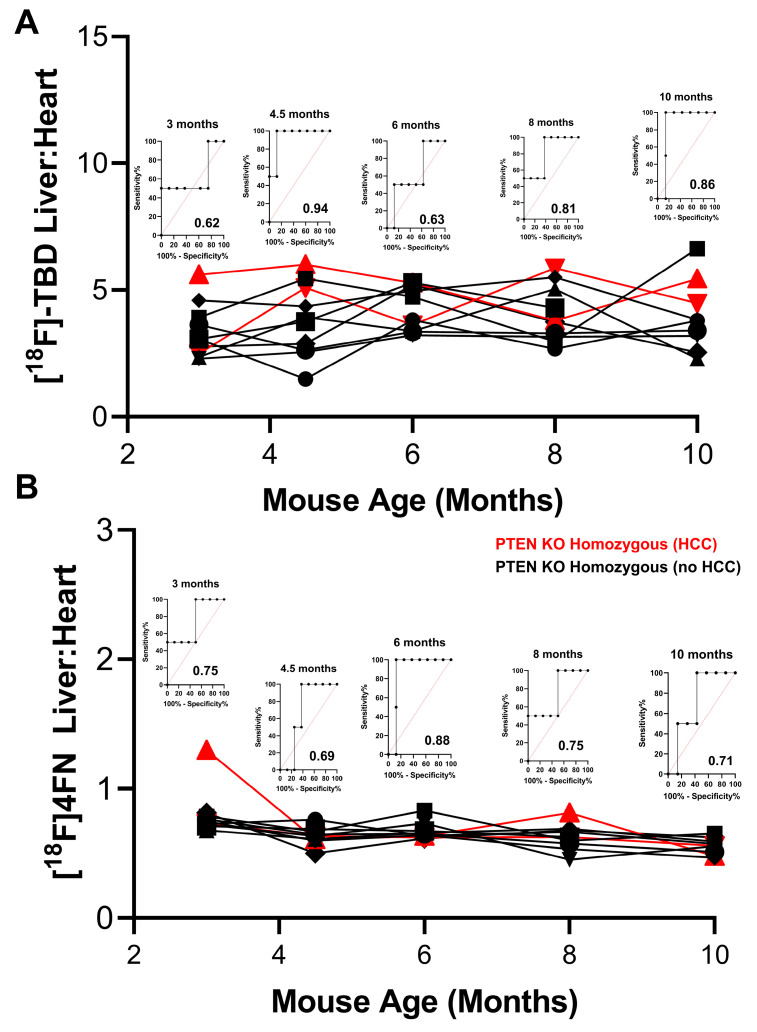
Liver uptake of ^18^F-TBD and [^18^F]4FN in female PTEN KO homozygous mice. (**A**) Liver:heart ratio of [^18^F]-TBD activity at 30 min post-injection at various time points (**B**) Liver:heart ratio of [^18^F]4FN activity at 60 min post-injection at various time points. Red = HCC progressor, black = HCC non-progressor. ROC analyses for HCC progression at each time point are inset above each time point.

The liver uptake of ^18^F-TBD and [^18^F]4FN was assessed in the female PTEN^−/−^ mice as described above (Figure 7). The liver uptake of ^18^F-TBD, as measured with the liver:heart at 30 min post-injection, remained relatively constant throughout the longitudinal study (Figure 7A). The increased uptake of ^18^F-TBD in the liver was predictive of eventual HCC progression as early as 4.5 months, analogous to the results seen in the male PTEN^−/−^ group. The increased ^18^F-TBD liver:heart was observed in HCC progressors at the 10-month timepoint, which was also in line with our observations in the male cohort. The [^18^F]4FN liver uptake in the female PTEN KO homozygous group was consistently low throughout the time course and showed minimal inter-animal variability (Figure 7B). This trend was highly reminiscent of that seen for the male PTEN^−/−^ group and again suggested that PTEN deletion in the liver suppresses the formation of high-energy ROS/RNS. The liver uptake of [^18^F]4FN at 6 months was modestly predictive for eventual HCC progression, but this tracer showed little predictive power at early and late time points. 

## 4. Discussion

In this study, we employed sequential multi-modal imaging with MRI, MRS, and PET/CT to quantify changes in the livers of mice in response to homozygous and heterozygous PTEN deletion. We chose to begin these imaging studies in juvenile mice (3 months of age) to capture early molecular and physiological predictors of eventual HCC progression. In the male cohort, homozygous PTEN deletion resulted in a dramatic increase in both liver fat and liver size relative to the PTEN^+/−^ controls, and the magnitude of this difference increased as the mice aged. Indeed, these data suggested that increased liver size and steatosis may manifest earlier than the 3-month time point, although follow-up experiments are required to bear this out. PTEN deletion is known to increase lipid synthesis in this model [28], and it appears to occur very early in the life cycle of the mouse. In contrast, homozygous PTEN deletion appeared to exert a much more modest effect on both liver size and fat content in the female cohort. Although female PTEN^−/−^ mice showed modestly increased liver steatosis relative to their male PTEN^+/−^ counterparts throughout the study, this imaging biomarker remained consistently below that seen for the male PTEN^−/−^ mice. Similarly, liver volume in the PTEN^−/−^ females remained in the normal range from 3 to 8 months, rising only in the 8- to 10-month period. Even with this increase, the liver size in the PTEN^−/−^ females remained consistently lower than the corresponding male cohort throughout the study. 

Although liver size and fat content were highly predictive for HCC progression in males (AUROC = 1 at all time points), they were relatively poor predictors for HCC progression in females. Liver fat was somewhat predictive for HCC progression in the female cohort at very early (3 months) and very late (10 months) time points, but the liver volume was unpredictive until the end of the time course, where HCC tumors were already quite large. Unfortunately, even the modest predictive value for liver fat in the female cohort may disappear in the context of benign fatty liver disease (e.g., NAFLD), which is likely to be a confounding factor in any HCC screening population. The parallel measurement of liver fat using CT, while again highly predictive in males, was even less predictive for HCC progression in the female cohort. Taken together, these results suggest that increases in steatosis may be less dramatic (and therefore less predictive of HCC) in the female population. The cost and technical complexity of MRS measurements further erode the feasibility of employing this imaging biomarker in screening protocols.

The increased uptake of the radiolabeled caspase 3 substrate ^18^F-TBD in PTEN^−/−^ males was observed early in the disease time course and was highly predictive of eventual HCC progression in this cohort. This observation was reproduced in the female cohort, although the effect size was less dramatic. Interestingly, subsequent histopathology revealed no PTEN-specific changes in liver apoptosis in either males or females between 3 and 4.5 months of age. This suggested that the differential uptake of ^18^F-TBD was not related to changes in the apoptotic rate between PTEN^−/−^ males and PTEN^+/−^ males, nor was it correlated with changes in the apoptotic rate between female PTEN^−/−^ HCC progressors and female PTEN^−/−^ HCC non-progressors. 

It is possible that ^18^F-TBD, while highly selective for the executioner caspases, may also be reporting on non-apoptotic caspase activity. For example, increased activity of the NRLP3 inflammasome—which facilitates the processing of IL-1β with caspase 1—is strongly associated with NAFLD, NASH, and HCC carcinogenesis [29]. Although ^18^F-TBD is a preferred substrate for caspase 3, our previous work suggested that it is also cleaved by caspase 1 [22], and it is possible that this off-target activity may be responsible for its enhanced liver uptake in the PTEN homozygous knockout mice. 

Alternatively, enhanced ^18^F-TBD uptake may instead be explained by altered transporter expression in the liver, which has been documented in mouse models of both NASH [30,31] and HCC [32]. The increased influx of the radiotracer due to overexpression of the organic anion transporters mediating liver uptake (e.g., OCT1, OAT2, OATP1B1, or OATP2B1) or reduced expression of transporters mediating efflux into the bile (e.g., P-gp, MRP2, MATE1, BCRP, or BSEP) could result in enhanced ^18^F-TBD liver uptake. Indeed, the radiotracer ^99m^Tc-mebrofenin showed increased hepatic exposure in NASH patients compared to healthy controls due to reduced biliary clearance, likely due to reduced activity of the MRP2 bile efflux transporter [33]. Altered expression and/or activity of liver transporters has been observed in human liver pathologies, including NASH [34] and HCC [35], and we believe that this phenomenon may play a role in the observed behavior of ^18^F-TBD. Follow-up experiments to assess transporter expression and activity in this model are ongoing.

Non-resolving inflammation and immune infiltration have been linked to NAFLD, NASH, and HCC, and they are implicated as a mechanism of HCC tumorigenesis [36,37]. Increased oxidative stress is associated with lipid metabolism in both NAFLD and NASH [38], and the infiltration of macrophages and neutrophils can result in an upregulation of myeloperoxidase (MPO) activity and other sources of high-energy ROS/RNS, particularly in humans and porcine models. In this study, we employed [^18^F]4FN to detect the presence of high-energy ROS/RNS in the liver, which would be indicative of both endogenous oxidative stress as well as neutrophil and macrophage activity. Surprisingly, [^18^F]4FN showed almost no liver retention in PTEN^−/−^ males at any time point. In contrast, liver retention of [^18^F]4FN in the PTEN^+/−^ males was highly variable throughout the study, exceeding liver uptake relative to PTEN^−/−^ males by two-fold at one point. [^18^F]4FN liver retention in the PTEN^−/−^ females was similarly depressed throughout the study with median liver:heart ratios below that of the PTEN^−/−^ males. The histological analysis of MPO and CD11b/Ly6 corroborated this image-based observation and suggested that high-energy ROS/RNS were suppressed. While the PTEN knockout mouse is known to progress through the production of high fluxes of low-energy ROS like H_2_O_2_ at 2.5 months [19], it remains unclear whether high-energy ROS/RNS are generated at later developmental stages in the PTEN knockout model, although this study suggests that they are not. Indeed, we note a strikingly low [^18^F]4FN liver uptake when HCC tumors are highly penetrant (Figure 4B). This could be explained using previous data indicating that PTEN activity inhibits the polarization of infiltrating macrophages from M1 to M2 [39,40]. This implies that the homozygous deletion of PTEN in the liver creates a “field effect” resulting in predominantly M2 macrophages and the suppression of high-energy ROS/RNS in the liver.

## 5. Conclusions

We have successfully carried out a 7-month, longitudinal, multi-modal imaging study of the PTEN deletion in an HCC mouse model and have obtained quantitative PET, MR, and CT imaging biomarkers as a function of disease progression. During this study, each mouse was sequentially imaged five times with two novel PET radiotracers, with no imaging-related adverse effects. This speaks to both the safety of these radiotracers in murine models as well as the optimized automated processes used to obtain them with a high radiochemical yield and purity. In almost every imaging modality, the effects of PTEN deletion in the liver were profoundly dependent on gender. The gender-dependent asymmetry of NASH and NAFLD prevalence has been well-documented in the clinical literature with NAFLD/NASH being more common in young men versus young women [41,42]. Similarly, HCC is more prevalent in males [43]. These trends appear to be linked to the presence of circulating estrogens, particularly estradiol, in pre-menopausal women [44], which may act to lower hepatic lipid accumulation, modulate the inflammatory response, and protect hepatocytes from cell death. The relatively mild steatosis in the female cohort may be correlated to the low penetrance of HCC resulting from PTEN deletion, although this needs to be substantiated by additional studies.

Based on the results of this study, additional investigation into the underlying mechanisms of sex-dependent differences in the PTEN deletion model may be required to support clinical translation. Because sex-based phenotypes in mice may either be private to rodents or conserved in humans [45], it was important to first demonstrate that the “imaging phenotypes”, like the highly sex-dependent emergence and penetrance of HCC in the liver-specific PTEN model, might also demonstrate sex-based differences. Indeed, MRI and CT (immediately clinically actionable), [^18^F]-4FN (actionable under IND), and ^18^F-TBD (potentially actionable) demonstrated imaging phenotypes consistent with sex-based differences in humans and HCC tumor development in the mice. Given the conservation of the imaging phenotype, future studies to determine the underlying root causes of the sex differences could be studied, leveraging well-reviewed and documented study designs [46,47]. Such experiments might include castration with and without hormone replacement in the male mice to determine the role of hormone-related gene regulation vs. other sex-linked genes. Alternatively, one could employ sex-hormone-specific knockouts on the background of liver-specific PTEN knockouts to identify the contribution of each hormone on the observed phenotype. While easily conceptualizable, these experiments are quite complex and time-consuming, and they are beyond the scope of the current study. From the perspective of clinical translation and by minimizing the use of animals in translation, the current data combined with the wide margin of safety for cGMP-produced ^18^F PET tracers may be sufficient to warrant the pilot testing of these radiopharmaceuticals in humans without further study of the root causes of these sex-based differences in rodents.

While almost all imaging biomarkers were highly predictive for HCC progression in the male cohort, only liver steatosis and ^18^F-TBD uptake provided any predictive power for HCC in the female cohort. An uptake of ^18^F-TBD in both males and females may result from early changes in the liver transporter expression prior to conversion to the malignant HCC phenotype. If so, this tracer may provide a sex-independent imaging biomarker for subsequent HCC screening workflows. Interestingly, [^18^F]FDG failed to provide predictive value in the male cohorts despite previous reports suggesting the up-regulation of glycolysis and glycolytic enzymes (pyruvate kinase M2 and hexokinase 2) in the PTEN liver deletion model [48]. Statistically, the detectable suppression of [^18^F]4FN uptake may be associated with immune suppression in the liver or the composition of the murine immune system, and early variability in the male heterozygotes may warrant further study. Overall, these studies illuminate the role of gender in the development of HCC in the PTEN liver model and suggest new roles for molecular imaging in HCC prediction.

## Data Availability

The data presented in this study are available from the corresponding authors upon reasonable request. Further individual data points are presented in key figures to facilitate data availability to the reader.

## Supplementary Materials

The following supporting information can be downloaded at https://www.mdpi.com/article/10.3390/cancers15153787/s1, Figure S1: radiosynthesis of ^18^F-TBD; Figure S2: liver density using CT and liver volume using PET (PTEN KO males); Figure S3: H&E staining of male PTEN KO livers; Figure S4: cleaved caspase 3 staining of male PTEN KO livers; Figure S5: TUNEL staining of male PTEN KO livers; Figure S6: MPO staining of male PTEN KO livers; Figure S7: CD11b/Ly6 staining of male PTEN KO livers; Figure S8: liver density using CT and liver volume using PET (PTEN KO females).
