# Supplementary material for: Gender Differences in a Mouse Model of Hepatocellular Carcinoma Revealed Using Multi-Modal Imaging
Source: Cancers (Basel). 2023 Jul 26;15(15):3787. doi: 10.3390/cancers15153787 (PMC10417617; doi:10.3390/cancers15153787)
Supplement: Supplementary file 1 [file cancers-15-03787-s001.zip › cancers-2421971-supplementary.pdf]

Supplementary Materials

Gender Differences in a Mouse Model of Hepatocellular Carcinoma Revealed Using Multi-Modal Imaging

Brian J. Engel [1], Vincenzo Paolillo [2], Md. Nasir Uddin [1], Kristyn A. Gonzales [3], Kathryn M. McGinnis [3], Margie N. Sutton [1], Madhavi Patnana [4], Brian J. Grindel [1], Gregory J. Gores [5], David Piwnica-Worms [1], Laura Beretta [3], Federica Pisaneschi [1,6], Seth T. Gammon [1,*] and Steven W. Millward [1,*]

1. Department of Cancer Systems Imaging, UT MD Anderson Cancer Center. Houston, TX
2. Cyclotron Radiochemistry Facility, UT MD Anderson Cancer Center. Houston, TX
3. Department of Molecular and Cellular Oncology, UT MD Anderson Cancer Center. Houston, TX
4. Department of Abdominal Imaging, UT MD Anderson Cancer Center. Houston, TX
5. College of Medicine, The Mayo Clinic. Rochester, MN.
6. Center for Translational Cancer Research, The Brown Foundation Institute of Molecular Medicine for the Prevention of Human Diseases (IMM) at The University of Texas Health Science Center at Houston, Houston, TX.

* Correspondence to Steven Millward (smillward@mdanderson.org) and Seth Gammon (stgammon@mdanderson.org)

Table of Contents

Supplemental Figure S1: Radiosynthesis of ^{18}F-TBD……………………………………………...…………S3

Supplemental Figure S2: Liver Density by CT and Liver Volume by PET (PTEN KO Males)…....………S4

Supplemental Figure S3: H&E Staining of Male PTEN KO Livers……………………………….…………S5

Supplemental Figure S4: Cleaved Caspase 3 Staining of Male PTEN KO Livers…………………….……S6

Supplemental Figure S5: TUNEL Staining of Male PTEN KO Livers ……………………………………S7

Supplemental Figure S6: MPO Staining of Male PTEN KO Livers ………………………………………S8

Supplemental Figure S7: CD11b/Ly6 Staining of Male PTEN KO Livers………..………………………S9

Supplemental Figure S8: Liver Density by CT and Liver Volume by PET (PTEN KO Females)……..….S10

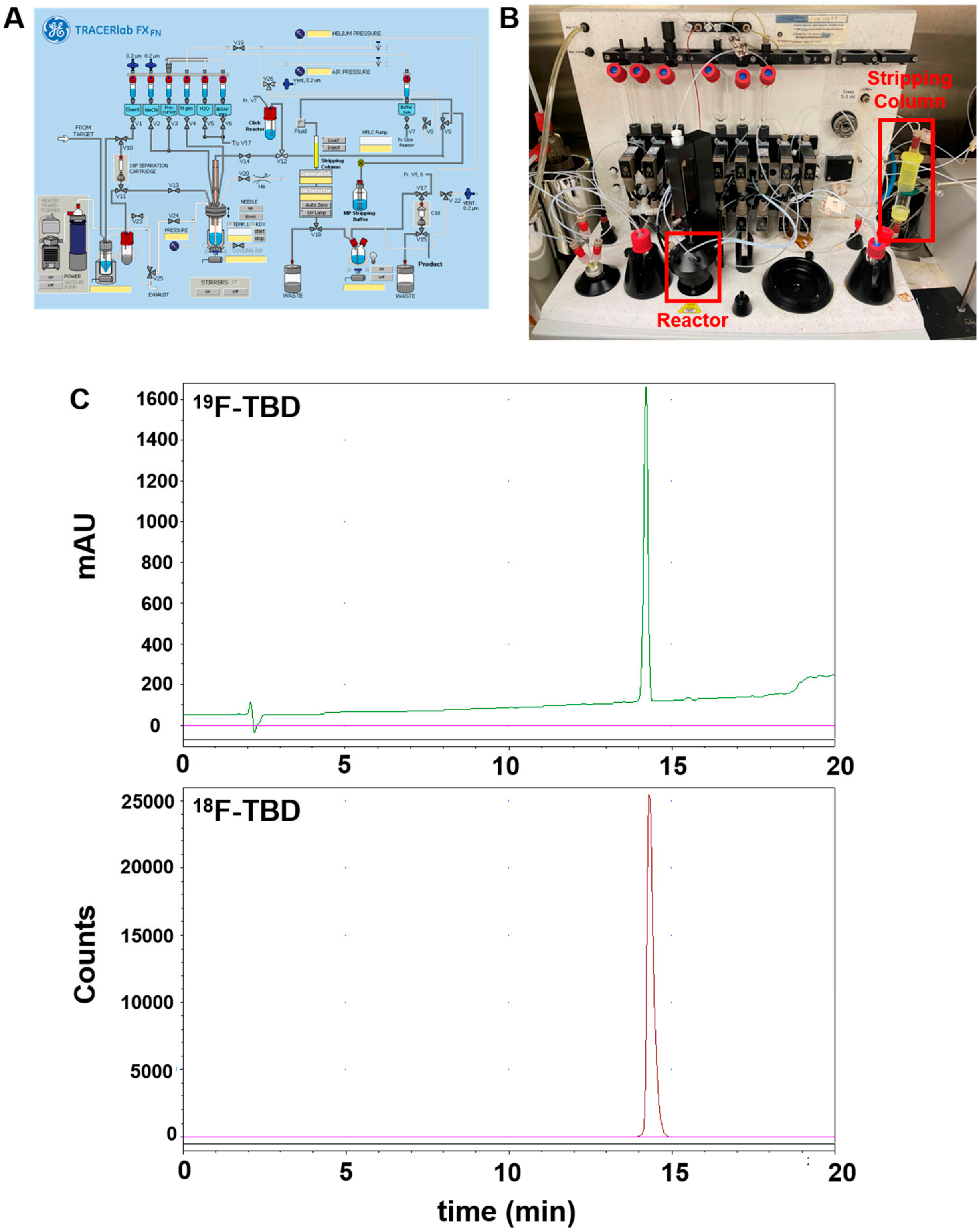

Supplemental Figure S1: (A) TracerLab Schematic for radiosynthesis of ^{18}F-TBD. **(B)** Photograph of partially configured TracerLab showing the location of the reactor and the stripping resin column. **(C)** C18 RP HPLC chromatogram of the ^{19}F-TBD cold standard (top) along with radioHPLC of purified and formulated ^{18}F-TBD.

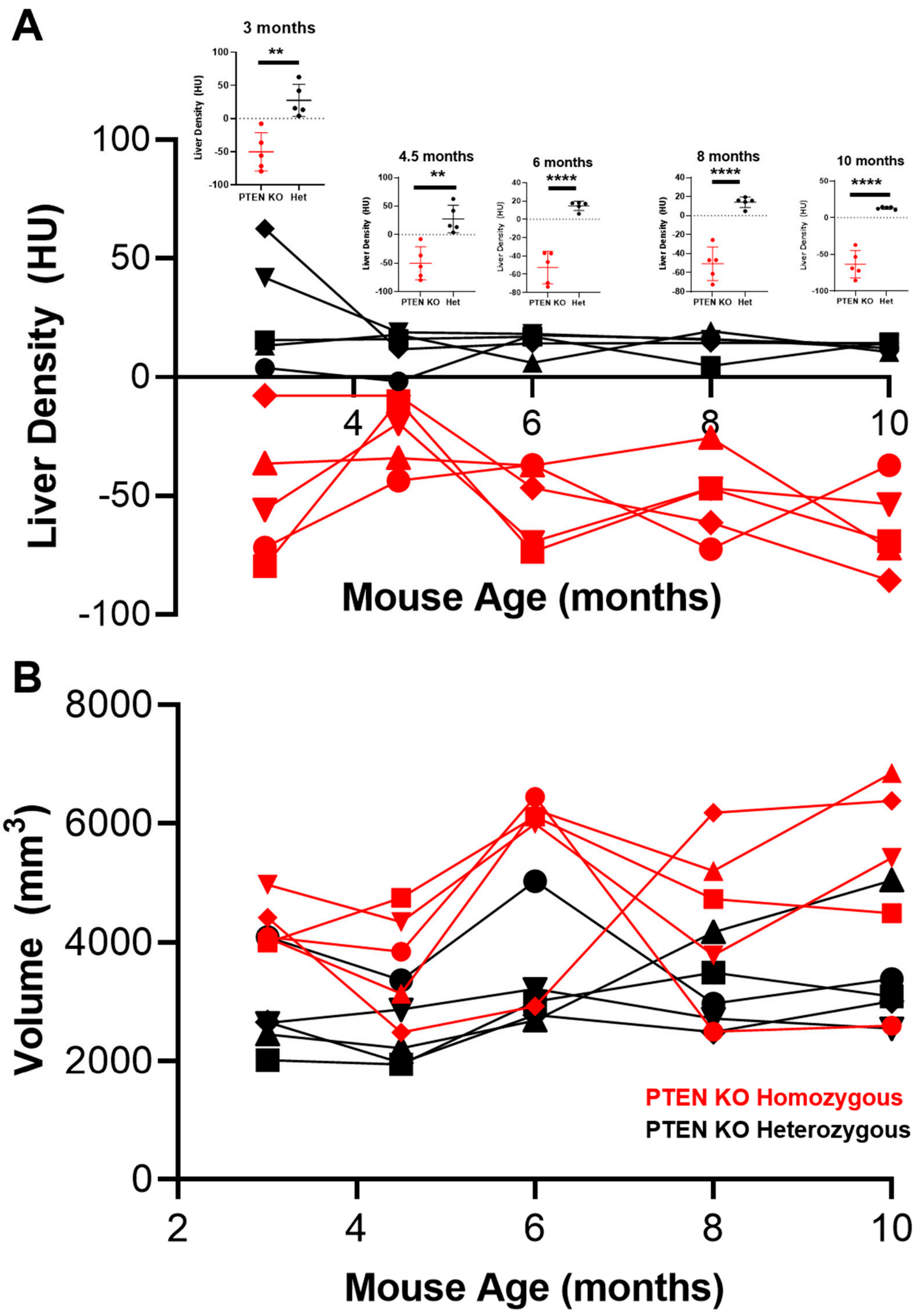

Supplemental Figure S2: (A) Liver Density by CT and **(B)** Liver volume by ^{18}F-TBD PET of PTEN$^{-/-}$ (red) and PTEN$^{+/-}$ (black) male mice over the timecourse of the longitudinal study.

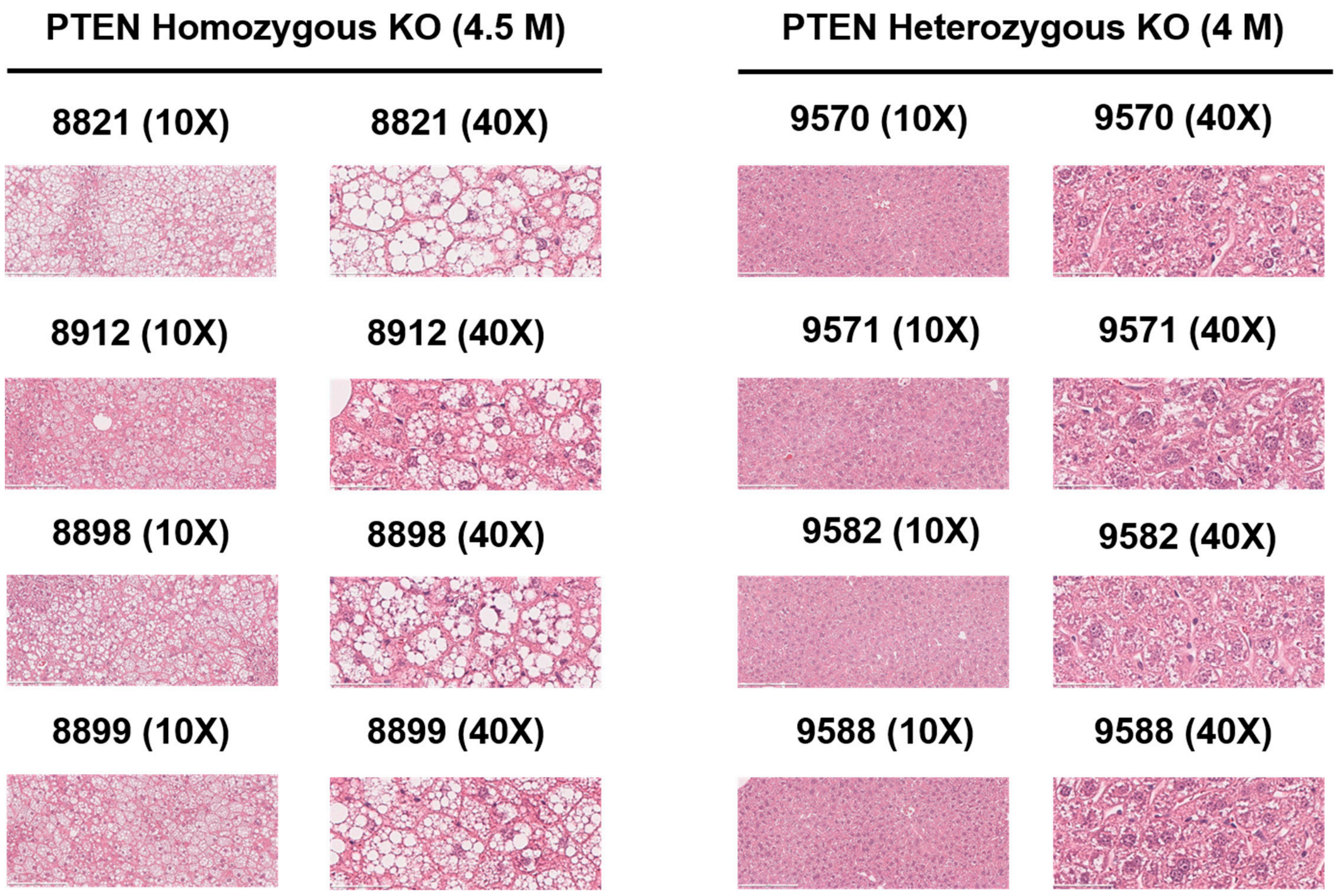

Supplemental Figure S3: H&E staining of livers from PTEN$^{-/-}$(PTEN Homozygous KO) and PTEN$^{+/-}$ (PTEN heterozygous KO) male mice 4–4.5 months of age. PTEN Homozygous KO livers show extensive steatosis as evidenced by the characteristic chicken wire appearance.

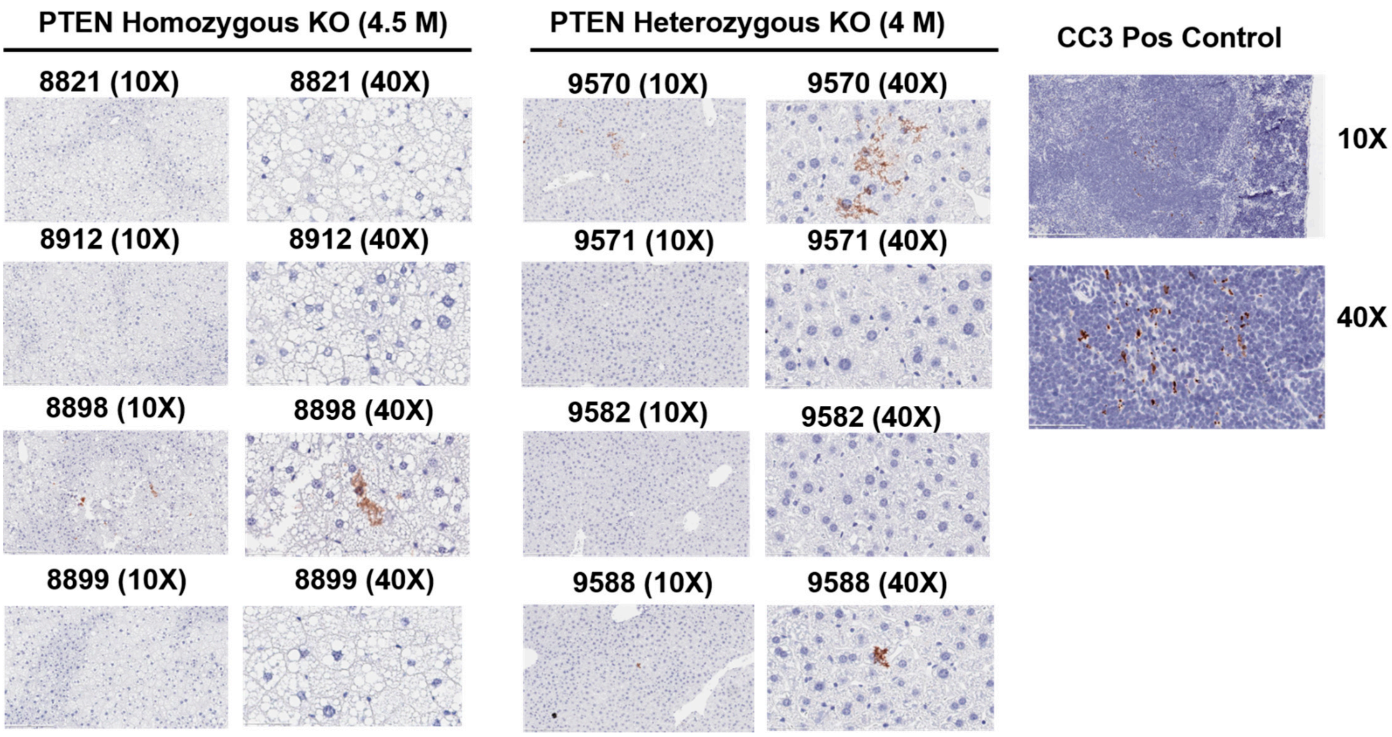

Supplemental Figure S4: Cleaved caspase 3 (CC3) staining of livers from PTEN$^{-/-}$(PTEN Homozygous KO) and PTEN$^{+/-}$ (PTEN heterozygous KO) male mice 4–4.5 months of age. A positive control showing cleaved caspase 3 staining in mouse spleen is shown on the right.

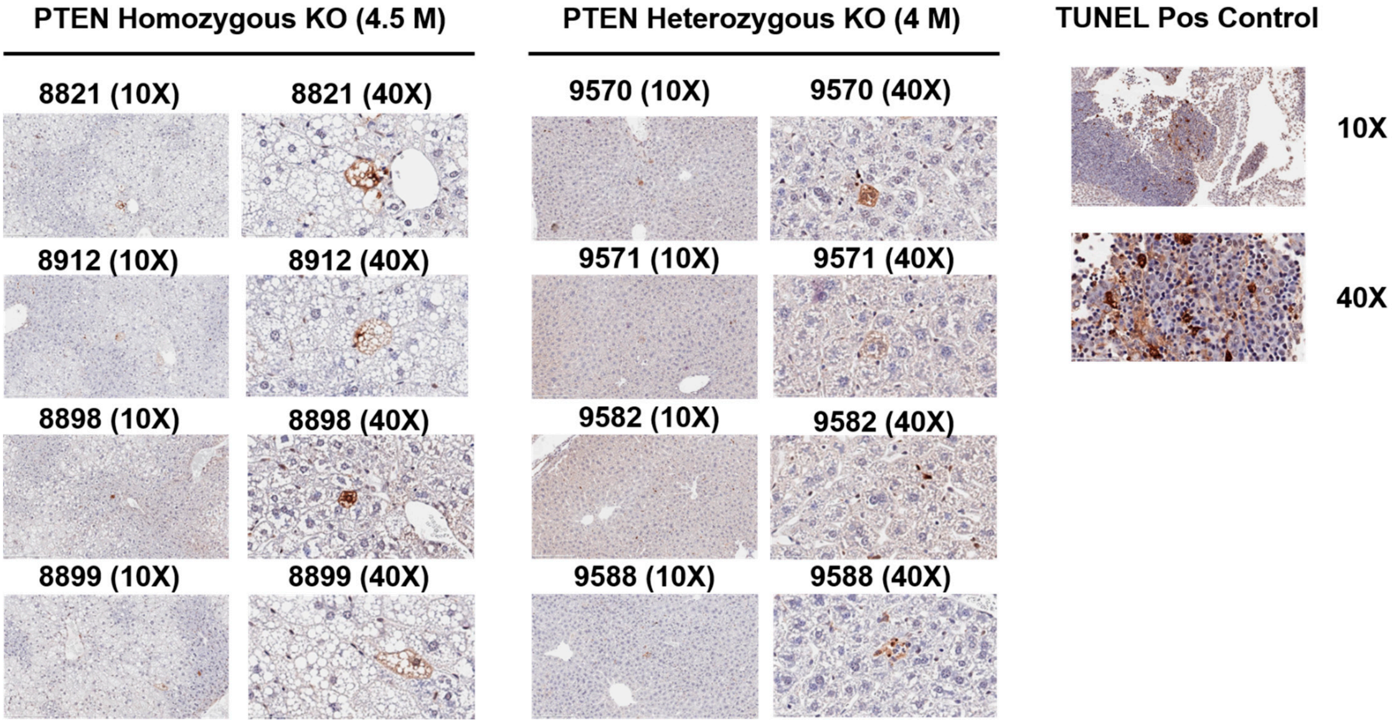

Supplemental Figure S5: TUNEL staining of livers from $PTEN^{-/-}$(PTEN Homozygous KO) and $PTEN^{+/-}$ (PTEN heterozygous KO) male mice 4–4.5 months of age. A positive control showing TUNEL staining in mouse embryo is shown on the right.

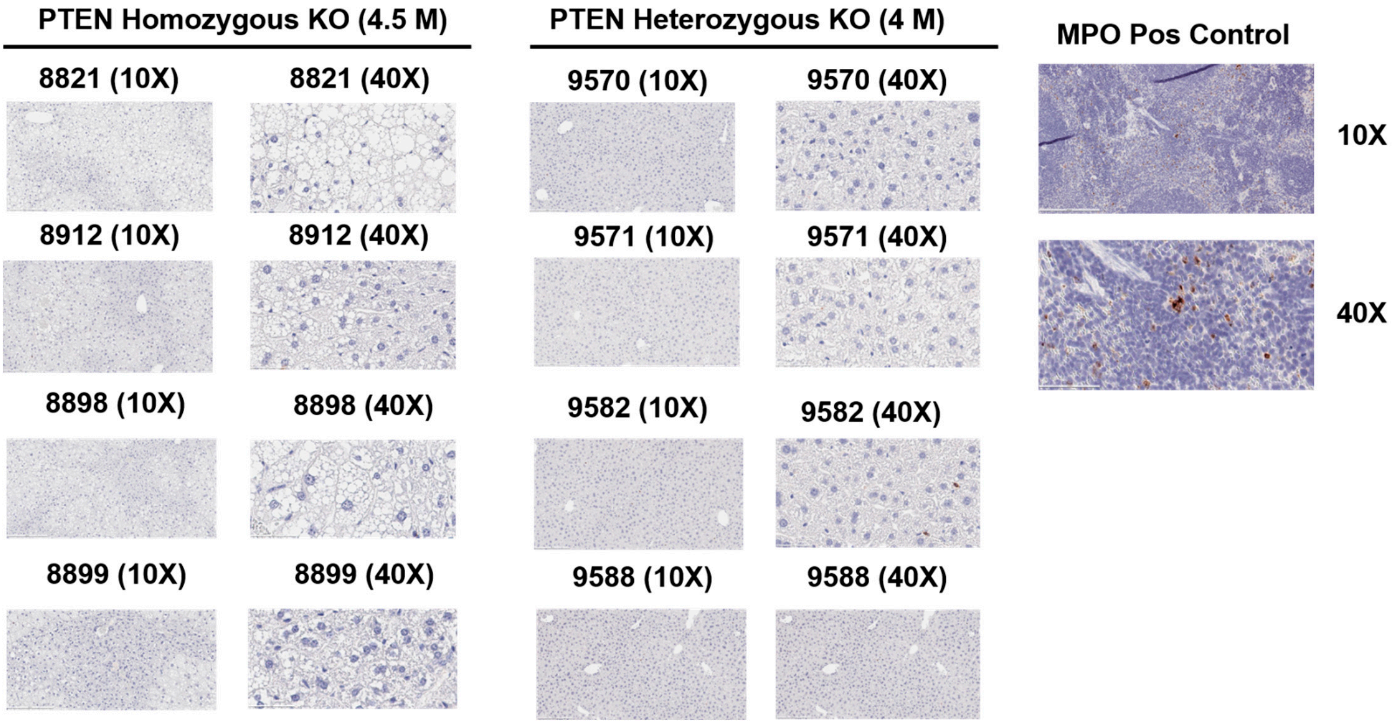

Supplemental Figure S6: Myeloperoxidase (MPO) protein level staining of livers from PTEN$^{-/-}$(PTEN Homozygous KO) and PTEN$^{+/-}$ (PTEN heterozygous KO) male mice 4–4.5 months of age. A positive control showing MPO staining in mouse spleen is shown on the right.

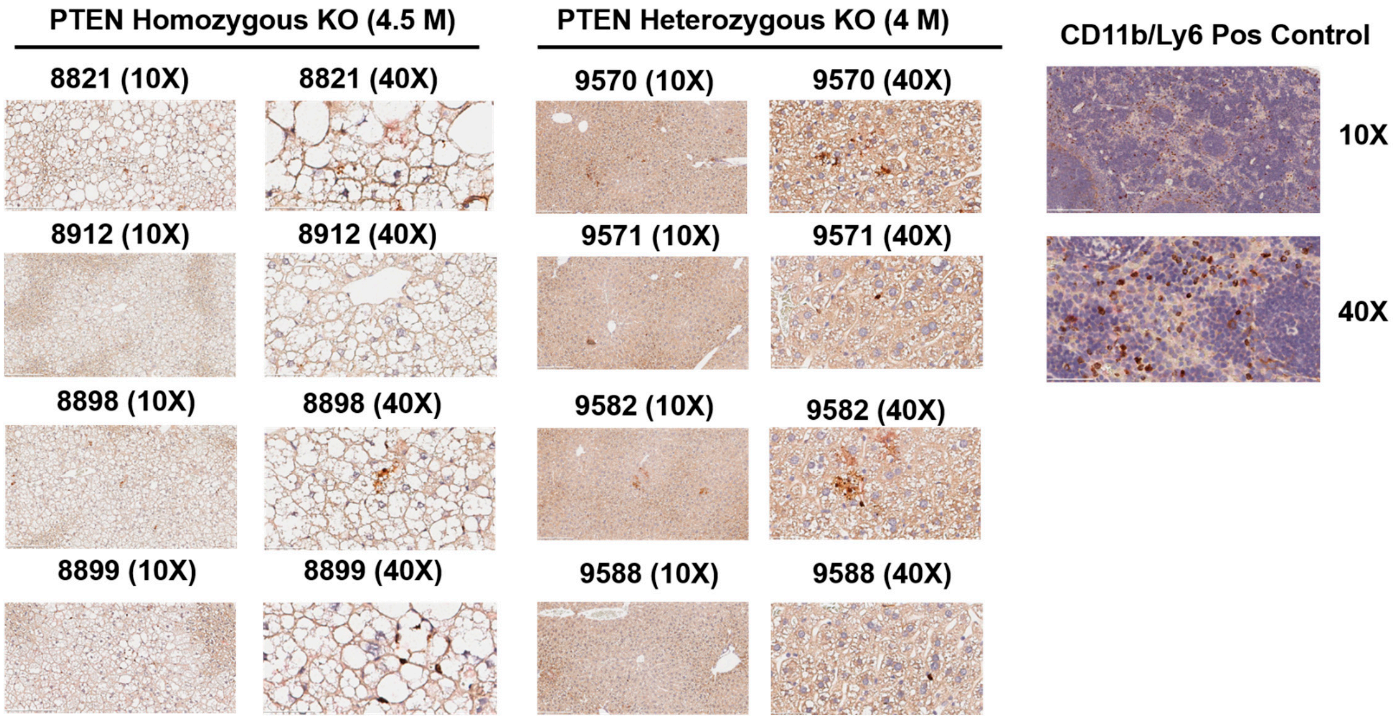

Supplemental Figure S7: CD11b/Ly6 staining of livers from PTEN$^{-/-}$(PTEN Homozygous KO) and PTEN$^{+/-}$ (PTEN heterozygous KO) male mice 4–4.5 months of age. A positive control showing CD11b staining in mouse spleen is shown on the right.

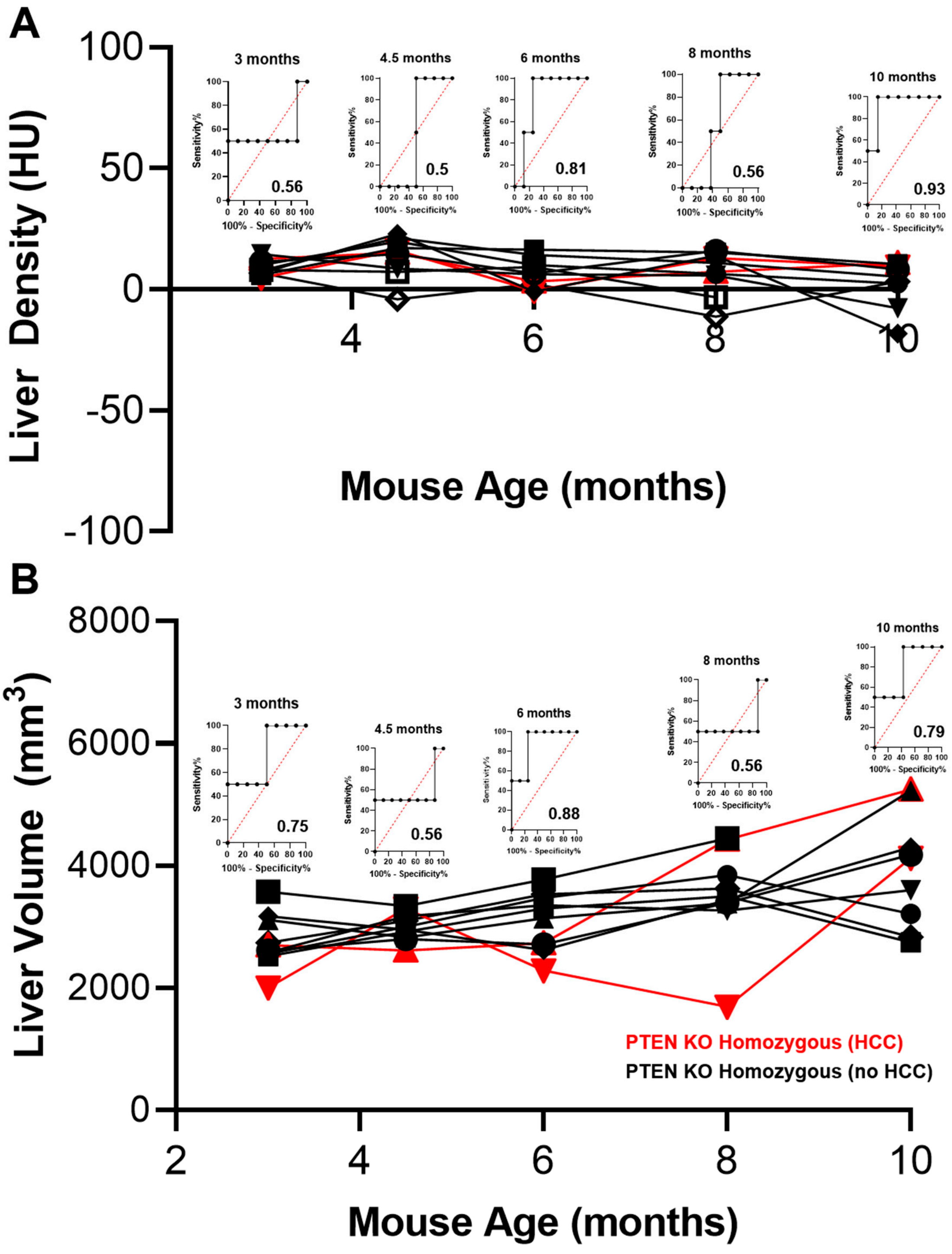

Supplemental Figure S8: (A) Liver Density by CT and **(B)** Liver volume by PET of PTEN$^{-/-}$female mice over the timecourse of the longitudinal study. ROC analyses for HCC progression point are inset above each time point.
